# Socio-economic factors do also matter: comments on the article “can climatic factors explain the differences in COVID-19 incidence and severity across the SPANISH regions?: an ecological study”

**DOI:** 10.1186/s12940-021-00702-5

**Published:** 2021-02-18

**Authors:** Pedro Muñoz Cacho, José L. Hernández, Marcos López-Hoyos, Víctor M. Martínez-Taboada

**Affiliations:** 1 Gerencia de Atención Primaria, Servicio Cántabro de Salud, Santander, Spain; 2 Department of Internal Medicine, Hospital Marqués de Valdecilla-IDIVAL, Santander, Spain; 3 Facultad de Medicina, Servicio de Reumatología, University of Cantabria, Hospital Universitario Marqués de Valdecilla, Avda. Valdecilla s/n, 39008 Santander, Spain; 4 Division of Immunology, Hospital Marqués de Valdecilla-IDIVAL, Santander, Spain; 5 Division of Rheumatology, Hospital Marqués de Valdecilla-IDIVAL, Santander, Spain

## Abstract

Phosri et al., commented on our previous study about the influence of climate variables at the beginning of the SARS-CoV-2 pandemic in Spain. They showed the impact of the association of gross domestic product (GDP) with the cumulative COVID-19 incidence per 10^5^ inhabitants in our country and the rise of several methodologic issues. Here we discussed the main advantages and disadvantages of ecological studies and we advocate to test the hypothesis created in this type of studies using individual-level research designs.

In response to the letter submitted by Phosri et al., concerning our paper [1], we agree with the authors that socioeconomic factors may influence the distribution of COVID-19 incidence. However, they surprisingly propose as a proof, the association of gross domestic product (GDP) with the cumulative COVID-19 incidence per 10^5^ inhabitants which, due to the lack of adjustment by any confounder factor, may be biased. This lack of controlling for possible confounders is the main criticism that they attribute to our study, that is, they do not apply any of the statistical methods which they propose.

Focusing on the variable “ultraviolet radiation” (UVR), there could be multiple possible confounder factors, some of them probably unidentified, and some others difficult to quantify. Among them, there are other meteorological variables, socio-economic factors, percentage of older adults, population density, presence of mass transit systems, incidence in neighboring populations, prevalence of highly transmissible variants, cultural and religious variables, etc. However, attempts to control for these variables in ecological studies do not prevent the persistence of the bias inherent to this type of epidemiological design [2–4].

Controlling confounders in ecological studies is more problematic than in individual-level studies. According to Harold Morgenstern *“even when all variables are measured accurately for all groups, adjusting for external risk factors may not reduce the ecological bias produced by these risk factors. In fact, it is possible that such ecological adjustment increases the bias”* [2]. Recently, Páez et al. [5] have suggested that an ecological study provides evidence that necessarily has to be verified with other research designs. A careful reading of this study [5] might also clarify some of the questions rised by Phosri et al.

In addition to the well-known ecological fallacy (or aggregation bias), ecological studies have other limitations, including the lack of adequate data, temporal ambiguity, collinearity, migration across groups, etc. For these reasons, ecological studies contribute to generate hypotheses but not to confirm them. In this sense, the hypotheses arising from ecological studies have to be evaluated in order to verify their biological plausibility, and those that have a sufficiently solid basis according to current knowledge should be contrasted using individual-level designs, such as cohort studies or clinical trials. In this line, in a case-control study, we have recently provided some evidence on the involvement of serum vitamin D levels (influenced by UVR exposure) in hospitalized COVID-19 patients [6]. Another Spanish group has carried out a pilot clinical trial with positive results of the efficacy of vitamin D supplements in reducing ICU admissions and mortality related to COVID-19 in hospitalized patients [7].

Finally, it should be pointed out that the statement made by Phrosri et al. *“Therefore, authors could not conclude that a whether variable, UVR predominantly influences COVID-19 incidence and severity unless other significant variables are included”*, does not reflect our interpretation of the results, as was clearly expressed in our original manuscript: *“Therefore, the design used serves to propose hypotheses that must be corroborated with other epidemiological designs”.*

Thus, to gain more knowledge about the causes explaining the greater transmissibility and severity of SARS-CoV-2 infection, it is necessary to use individual-level research designs, mainly randomized clinical trials, rather than trying to control for possible confounders in ecological studies.

## Data Availability

National official sources (Ministry of Health; https://www.mscbs.gob.es/) and Spanish Meteorological Agency (upon request: http://www.aemet.es/),
